# Mental privacy: navigating risks, rights and regulation: Advances in neuroscience challenge contemporary legal frameworks to protect mental privacy

**DOI:** 10.1038/s44319-025-00505-6

**Published:** 2025-06-25

**Authors:** Łukasz Szoszkiewicz, Rafael Yuste

**Affiliations:** 1 Faculty of Law and Administration and Cognitive Neuroscience Centre, Adam Mickiewicz University, Poznan, Poland; 2 NeuroTechnology Center, Columbia University, New York, NY USA

**Keywords:** Economics, Law & Politics, Neuroscience

## Abstract

Rapid advances in neurotechnology are eroding the boundary between mental activity and data, creating urgent risks for mental privacy. This article examines the regulatory landscape and proposes a framework – grounded in user agency, data solidarity, and precaution – to strengthen protections for neural data.

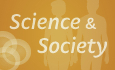

Rapid advances in neurotechnology—devices capable of recording or modulating the activity of the central or peripheral nervous system—are eroding the boundary between mental activity and data. Invasive brain implants can translate neural signals into intended movement, emotions, facial gestures or speech; high-resolution brain imaging enables effective decoding of emotions, language, mental imagery and psychological intent; non-invasive consumer devices measuring brain signals at the scalp can infer inner language, attention, emotion, sexual orientation and arousal among other cognitive functions. Vice versa, neurofeedback devices that enable to train mood or focus, or specific brain states by real-time monitoring of brain activity are now explored in clinical trials to enhance brain plasticity after trauma or disease.

“… non-invasive neurotechnology, such as EEGs, near-infrared imaging or portable brain scanners, headsets and bracelets, are increasingly entering an essentially unregulated consumer marketplace.”

While invasive neurotechnology, such as cortical implants, necessitates neurosurgery and therefore falls under medical regulations, non-invasive neurotechnology, such as EEGs, near-infrared imaging or portable brain scanners, headsets and bracelets, are increasingly entering an essentially unregulated consumer marketplace (Box 1). These rapid developments harbor the risk that intimate neural data are collected, analyzed and potentially misused. Contemporary legal frameworks offer only limited protection for such uniquely sensitive data, creating an urgent need for targeted safeguards to preserve mental privacy. We propose strengthening existing data protection regulations with a broad approach to safeguard neural data, as it is the coinage in which brain activity, which determines mental function, is written.

## Neurotechnology today: capabilities and risks

The current neurotechnology landscape encompasses a range of invasive and non-invasive devices capable of both decoding and modulating neural activity. Significant progress has for instance been achieved in brain-computer interfaces (BCIs), devices that connect brain activity to an external machine or computer in an open- or closed-loop fashion. For example, intracortical electrode implants in the motor cortex can decode desired arm movements to control robotic arms or cursors, even in completely paralyzed patients. Recent experiments involving motor control tasks demonstrate high accuracy, often exceeding 85% with sufficient training (Patrick-Krueger et al, 2025). Even greater advances have been reported in speech decoding. In 2024, researchers, using implanted sensors, successfully decoded attempted speech in individuals with amyotrophic lateral sclerosis (ALS), reaching accuracy levels as high as 97.5% (Card et al, 2024). Collectively, these studies show that invasive BCIs can translate mental activity into actions to restore communication and mobility for persons with disabilities. At the same time, such applications pose ethical and legal challenges regarding data privacy—for example, the possibility of “eavesdropping” on private verbal thought as reported by Kunz et al, 2024, or the re-identification of individuals from shared anonymized medical records—and security, as well as user agency, that is, providing volitional control over decoding (Sankaran et al, 2023).

At the other end of the spectrum, non-invasive methods that combine brain imaging and artificial intelligence now allow researchers to decode aspects of a person’s experiences directly from brain activity. For example, recent studies have trained AI systems to reconstruct visual imagery that a person has seen, using data from brain scans. In one demonstration, an AI model was able to generate recognizable pictures of objects—a teddy bear, an airplane, and so on—from fMRI brain scans recorded while participants viewed those images (Takagi and Nishimito, 2023). Beyond visual reconstruction, non-invasive decoders have even recovered continuous language from brain activity. Tang et al (2023), again by combining fMRI recordings with AI, could reconstruct continuous language from cortical semantic representations, producing intelligible word sequences that captured both the exact wording, and the overall meaning of stories participants had heard.

“… recent studies have trained AI systems to reconstruct visual imagery that a person has seen, using data from brain scans.”

This progress is not limited to laboratory settings but is entering the marketplace. Devices such as consumer-grade EEG headsets are gaining popularity owing to their portability and affordability. These detect brainwaves and “P300” responses to control smartphone applications, decode states such as attention, relaxation and basic emotions: happiness, sadness, anger, fear, disgust, and surprise (Houssein et al, 2022). These devices can achieve satisfactory accuracy, although with considerable variability between individuals (Caiado and Ukolov, 2025).

Current non-invasive BCIs have limited resolution, but research shows they can still reveal sensitive information. For example, by training on 175 h of EEG recordings, researchers correctly identified almost half of 512 spoken phrases, demonstrating that decoding accuracy improves as more data are used (Sato et al, 2024). Non-invasive neurotechnology is also increasingly being employed to diagnose certain mental health and neurological disorders, such as epilepsy (Peltola et al, 2023), further demonstrating the potential of consumer-grade devices to provide medically sensitive information about the user.

As consumer neurotechnology becomes ever more accessible, we can expect these devices—and the data they generate—to make their way into the workspace and society in general, just as other wearables have before them. This could, among many other places, impact courtrooms. The question of if and how data extracted from wearables should be admitted in criminal proceedings is already provoking intense debate among legal experts (Nicolai et al, 2024), underlining the urgent need to strengthen protections for the right against self-incrimination in the era of neurotechnology.

Box 1 Privacy practices of consumer neurotechnology companies Despite collecting medical-grade brain data, many consumer neurotech companies operate in a regulatory vacuum. To document this gap, the Neurorights Foundation, a US-based non-profit organization, published a report that reviewed user agreements of thirty direct-to-consumer companies. The analysis benchmarked each policy against six global privacy frameworks within five thematic areas: (1) access to information; (2) data collection and storage; (3) data sharing; (4) user rights; (5) data safety and security. The report’s findings expose significant inconsistencies between industry practices and global norms, including:

- All companies take possession of all the user´s neural data.
- Twenty-nine of the thirty companies retain unfettered rights to access consumers’ neural data.
- Most companies explicitly permit the sharing of neural data with third parties, often under broad and vaguely defined terms.
- Many companies fail to provide clear information about the neural data being collected, with some not even mentioning “neural data” explicitly in their privacy policies.
- No company adequately explains the sensitivity of neural data or the potential information that can currently be decoded from it.
- Provisions enabling users to withdraw consent, access their data, or request the deletion of neural recordings are inconsistently applied, if provided at all.
- Many companies demonstrate insufficient data security practices, lacking specific commitments to encryption, breach notification, or dedicated safeguarding of neural data.

## Why is mental privacy unique?

Mental privacy, defined here as the protection of the mental activity of an individual, presents distinct challenges beyond those of traditional data protection. Neural data are uniquely sensitive because they reveal our most intimate processes—thoughts, memories, mental states, emotions, behavior, personality and health conditions—and can even forecast future behavior, health risks or cognitive performance. Crucially, these signals also reflect subconscious and involuntary activity, exposing information that individuals may not consciously recognize.

“Neural data are uniquely sensitive because they reveal our most intimate processes […] and can even forecast future behavior, health risks or cognitive performance.”

From a human-rights perspective, intrusions on mental privacy may, in some instances, amount to a violation of the freedom of thought and conscience, an absolute right protected under international human rights law. If thought is inviolable, so too should be the data that can be used to infer it. Unlike conventional privacy, mental privacy demands a tailored regulatory framework. As it can reveal the most intimate aspects of a person, neural data must be treated as fundamentally distinct, with enhanced safeguards to uphold dignity, autonomy and mental integrity.

“If thought is inviolable, so too should be the data that can be used to infer it.”

For this reason, “neural data” has already been explicitly defined in emerging legal frameworks as information generated by measuring activity in the nervous systems, sometimes extending to inferences drawn from that data. This includes activity in the central nervous system—the brain and spinal cord—as well as the peripheral nervous system—the neural ganglia and nerves throughout the body. Crucially, neural data is not limited to electrical brainwaves. It encompasses any measurable signal that reflects neural activity, whether obtained electrically, chemically or via other means. A direct example is the action potential or synaptic currents detected by electrodes, such as the signals captured by electrical implants or EEG-based devices. But neural activity can also be measured indirectly through physiological proxies: for instance, fMRI scan maps blood flow changes in the brain, which are an indicator of neural activation. Other neurotechnologies use optical, magnetic or biochemical sensors to measure indirectly neuronal activity by the effect that said activity has on its environment. Some peripheral biosignals should also fall under the category of neural data: for example, muscle nerve signals (EMG) reflecting intended movements, as the muscle fibers faithfully respond to the activity of spinal cord motoneurons. Such technologies are increasingly incorporated into both medical devices such as Synchron’s minimally invasive intravascular electrodes that measure motor signals or consumer products, for instance, Meta’s EMG-based neural wristband.

## Current regulatory landscape

Neural data, defined here as any data collected by neurotechnological devices, are not classified as sensitive information when collected outside medical contexts by consumer products. This regulatory gap has been increasingly acknowledged at both national and international levels.

Binding legal protections first emerged in Latin America with Chile’s pioneering 2021 constitutional amendment, which protects “cerebral activity and the information drawn from it”—that is, brain data—as a constitutional right. This amendment led to a 2023 unanimous ruling by Chile’s Supreme Court ordering a company to delete a consumer’s neural data, as its collection violated mental privacy protections. Inspired by the Chilean example, a similar constitutional amendment was approved in 2023 in the Brazilian state of Rio Grande do Sul and legislators in Ecuador, Colombia, Mexico and Uruguay have already introduced bills to address similar concerns.

In the USA, several states have moved to define “neural data” within their privacy legal frameworks and extend it heightened protections as sensitive personal information. Colorado, California and Montana now explicitly include neurotechnology and neural data in their statutory definitions, and apply to these data existing consumer legislation for personal sensitive data. Other states like Alabama, Connecticut, Massachusetts, Minnesota, Illinois and Vermont are considering similar legislation aimed at protecting neural data. Moreover, three US Senators have also recently requested the US Federal Communications Commission (FCC) to examine practices of the neurotechnology industry with respect to neural data. It is worth noting that all legislative efforts worldwide have been unanimous or nearly unanimous, reflecting emerging bipartisan agreement and a growing recognition of the need for tailored protections grounded in medical and scientific consensus definitions.

Besides these legislative processes, non-binding standards, so-called “soft law” that serve as ethical guidelines for neurotechnology, are taking shape at the national and international level. The OECD issued its first neurotechnology guidelines in 2019, and the Organization of American States’ Inter-American Juridical Committee followed with recommendations in 2021 and 2023. UNESCO published a draft instrument in 2024, currently under intergovernmental negotiation. In 2025, the UN Special Rapporteur on the right to privacy urged all states to enact specific regulatory regimes for neurotechnologies and neural data, given their profound implications for privacy, dignity and fundamental rights. Most importantly, the UN Human Rights Council Advisory Committee recommended developing General Comments on the freedom of thought and mental integrity for persons with disabilities. General Comments offer authoritative interpretations of treaty obligations and, once adopted, may be invoked before national courts and international bodies, paving the way for a global human-rights regulation of neural activity and data.

In Europe, both the Council of Europe and the European Union have produced expert reports on neurotechnology, but the privacy standards set by the EU’s General Data Protection Regulation has so far obviated the need for standalone legislation. GDPR’s definitions of personal and sensitive data—and its enforcement mechanisms—already offer stronger safeguards for neural data than those in many other jurisdictions. Nevertheless, neurotechnology is not explicitly mentioned in the GDPR, crafted more than a decade ago, and whether it applies to this novel area is a subject to debate. Also, certain GDPR provisions, particularly those governing purpose limitation and informed consent, may warrant refinement to address the specific challenges posed by neural data (Istace, 2024). On the other hand, the recently adopted EU’s AI Act classifies AI-based neurotechnology that uses “significantly harmful subliminal manipulation” as prohibited, further reinforcing mental privacy safeguards (European Commission, 2025). The AI Act also prohibits the use of AI systems to infer emotions in the workplace and educational institutions, unless it serves a clearly defined medical or safety purpose.

Aside from a universal concern to protect the privacy of neural data, these varied instruments contribute to a growing consensus around core definitions and principles. “Neural data” is generally defined as information generated by measuring activity in the central or peripheral nervous systems, sometimes extending to inferences drawn from that data. Equally clear is the shared understanding that neural data demand targeted legal interventions and enhanced protections—particularly against non-consensual or purposefully manipulative uses.

## Agency, solidarity, precaution

Although these initial legal steps to safeguard mental privacy appear timely, we think that they may be insufficient, as three critical vulnerabilities persist that should be addressed.

First, users need to exert meaningful control over their own neural data. While the mentioned existing legislation can protect the governance of neural data, it falls short from providing the user agency on its collection and use. For example, consenting to data collection of a meditation headset in the morning automatically authorize the device to continue logging micro-fluctuations related to anxiety or attention later in the day. Yet, in many instances, the consumer has no practical way to inspect, amend or delete the data that have been captured. The capacity of neurofeedback to covertly influence individual preferences and mental associations presents a particularly troublesome risk of exploitation for political and commercial manipulation (Furnari et al, 2024). True agency requires that individuals retain ongoing, transparent control over their neural data—in device design, software features and governance frameworks alike.

Second, the circulation of neural data needs to be restricted, instead of driven by market forces. Consumer-grade headsets often stream raw brain signals or algorithmic inferences under wafer-thin consent, leaving users without appropriate information about how their data might be analyzed, sold, reused or even transferred abroad, outside any domestic safeguards. One hard-line solution would be to ban all sharing or trade of neural data. A more balanced alternative—echoing the concept of data solidarity—would treat neural data as a shared resource that serves the public good (Prainsack et al, 2022). Under this model, people who contribute their data would share in its benefits, while high-value research would receive public support in the form of financial or practical assistance.

Another practical alternative is to follow the medical model and consider all neurotechnology, including consumer-grade, as medical devices (Goering and Yuste, 2016). This would render all neural data medical and thus, protected by appropriate regulation already implemented in most countries. Moreover, as the UK Information Commissioner’s Office warns, consumer neurotechnology is poised to generate vast datasets essential for medical research, yet current legal frameworks may block their ethical reuse. A medical model could incorporate all neural data under the same umbrella, while data solidarity governance offers a path that both protects individual rights and unlocks neural data for socially beneficial purposes.

“Even where agency is protected and data solidarity frameworks are in place, neurotechnology still asks society to gamble with unknowns.”

Third, lawmakers should be guided by the precautionary principle, that is, assume potential risks. Even where agency is protected and data solidarity frameworks are in place, neurotechnology still asks society to gamble with unknowns. International human rights law enshrines this precautionary principle: when credible evidence suggests serious or irreversible harm, states should act to prevent that harm—despite scientific uncertainty (Donders and Plozza, 2023). Practical steps might include post-market surveillance of consumer EEG devices, mandatory human rights risk assessments, or temporary moratoria on the most controversial neurotechnologies until longitudinal data accrue. These measures should be developed in a deliberative process, which involves patients, scientists, policymakers, industry and the general public. Such proportionate, transparently reviewed measures guard against both premature bans and unchecked deployment, ensuring innovation proceeds without sacrificing mental privacy or human dignity.

## Conclusion: the last privacy frontier

Neurotechnology is advancing faster than our legal and ethical frameworks can adapt. Neural data are uniquely intimate and sensitive, and current privacy laws do not fully capture their special nature. Emerging scholarship and guidance provided by international human rights law emphasize that mental privacy should enjoy enhanced protection as it is intimately tied to freedom of thought while national and international regulations signal growing consensus that additional rules are needed. As the concerns related to increasingly accessible consumer-grade neurotechnology underscore the urgency of legislative response, it is equally important to avoid alarmist narratives or overhyping potential harms or regulatory overreach. A measured, evidence-driven approach will best serve the development of balanced legal frameworks that protect from harm and exploitation, and implement the principles of user agency, solidarity and precaution. Failure to act now risks eroding the last private frontier—the mind itself—at a time when neuroscience and neurotechnology is poised to unlock it.

“Failure to act now risks eroding the last private frontier—the mind itself—at a time when neuroscience and neurotechnology is poised to unlock it.”

## Supplementary information

Peer Review File
