# Supplementary material for: Mental privacy: navigating risks, rights and regulation: Advances in neuroscience challenge contemporary legal frameworks to protect mental privacy
Source: EMBO Rep. 2025 Jun 25;26(14):3469–73. doi: 10.1038/s44319-025-00505-6 (PMC12287510; doi:10.1038/s44319-025-00505-6)
Supplement: Supplementary file 1 — Peer Review File [file 44319_2025_505_MOESM1_ESM.pdf]

EMBO reports

Mental Privacy: Navigating Risks, Rights and Regulation

Lukasz Szoszkiewicz and Rafael Yuste

Corresponding author(s): Lukasz Szoszkiewicz (l.szoszkiewicz@amu.edu.pl)

Review Timeline:

Submission Date:	15th May 25
Accepted:	11th Jun 25

Editor: Holger Breithaupt

Transaction Report: This article was editorially reviewed.
